# Thermal Dynamics Effects using Pulse-Shaping Laser Sintering of Printed Silver Inks

**DOI:** 10.1038/s41598-018-19801-4

**Published:** 2018-01-23

**Authors:** M. Bolduc, C. Trudeau, P. Beaupré, S. G. Cloutier, P. Galarneau

**Affiliations:** 1 Institut National d’Optique, 2740 Einstein Street, Québec, QC G1P 4S4 Canada; 2 Department of Electrical Engineering, École de Technologie Supérieure, 1100 Notre-Dame Ouest, Montréal, QC H3C 1K3 Canada

## Abstract

In recent years, additive manufacturing has been evolving towards flexible substrates for the fabrication of printable electronic devices and circuits. Generally polymer-based, these emerging substrates suffer from their heat sensitivity and low glass-transition temperatures. As such they require new highly-localized sintering processes to treat the electronic inks without damaging the polymer-based substrate. Laser-based sintering techniques have shown great promises to achieve high-quality sintering locally, while controlling the heat penetration to preserve the polymer substrates integrity. In this report, we explore new optimization pathways for dynamic laser-based sintering of conductive silver inks. Multiple passes of a pulsed laser are first performed while varying pulse train frequencies and pulse energies as an attempt to optimize the properties of the silver inks. Then, time-domain pulse shaping is performed to alter the properties of the conductive inks. Together, these pathways allow for the careful control of the time-domain laser energy distribution in order to achieve the best electronic performances while preserving the substrate’s integrity. Sheet resistance values as low as 0.024Ω/□ are achieved, which is comparable to conventional 1-hour oven annealing, with the processing time dramatically reduced to the milisecond range. These results are supported by finite element modeling of the laser-induced thermal dynamics.

## Introduction

The field of Printed Electronics has grown dramatically in the recent years, with applications ranging from photovoltaic devices^1^ to printed organic field-effect transistors^2,3^ and printable OLED displays^4^. The assembly of complex multilayered device architectures proves highly challenging as it requires the carefully-controlled deposition of precise layers of various types of materials in printable ink solutions atop each-other. This is often especially challenging due to basic compatibility issues between the inks and/or substrates, sensitivity to the processing environment and thermal effects. Indeed, these electronic inks usually require thermal annealing to produce highly-functional solid materials. Although conventional thermal furnace annealing is still the standard in today’s industry, a move towards rapid optical processing is necessary for high-speed mass production and integration with flexible temperature-sensitive polymer-based substrates. Recently, research efforts explored highly-localized thermal treatments using infrared and ultraviolet lamps^5,6^, high power flash lamps^7–12^, and continuous-wave laser irradiation^13–19^ have shown great promise for achieving control of the final ink properties.

More recently, it was shown that microsecond-pulsed lasers allow careful control of the irradiation to reach thermal equilibrium for the different materials present in ink solutions^20,21^. This aspect is critical as the heterogeneous nature of these printable inks leads to complex heating dynamics and phase transitions, including the evaporation of different organic solvents and surfactants with the sintering of nanoparticles. As such, microsecond-pulsed lasers can provide rapid and highly-localized annealing of the inks without thermal damage to the flexible polymer substrates, while providing a wider range of temperature sensitive inks^17–19,22^. So far, pulsed laser technology has been used mostly with digital inkjet and aerosol-jet printing technologies, where local laser sintering paths can be adapted as quickly as the design layouts to allow rapid device prototyping^23,24^.

In this paper, we show that controlling the incident laser pulse’s energy distribution in the time-domain is paramount to optimizing sintering process in heterogeneous nanoparticle-based conductive silver inks. A multi-step microsecond-pulsed laser process and a time-domain pulse-shaping modulation have been successfully used to target specific phase transitions in the sintering of silver inks and yield better uniformly-annealed conductive printed traces. As we show, the solvent’s boiling point is the key parameter to implement this approach for a specific nanoparticle-based ink. For low boiling-point solvents such as water, the difference in the temperatures needed to both evaporate the solvent (~100 °C) and melt the metallic nanoparticles (~200 °C) is somewhat large. As such, a two-step method is required to both evaporate the solvent and sinter the nanoparticles. For higher boiling-point solvents such as Ethylene or Polyethylene Glycol (150–255 °C), the solvent evaporation point is closer or above the temperatures needed to melt the metallic nanoparticles. There, a carefully-tuned one-step process using time-domain pulse modulation works best. Finite-element method (FEM) simulation of the thermal effects occurring during laser-mater interactions with time-domain pulse-shaping modulation is consistent with our experimental observations that controlling the heating rate for the sintering process gives rise to improved conductivity for the printed silver nanoparticle-based inks.

## Results

### Ag Ink Resistivity Control

**Figure 1:**
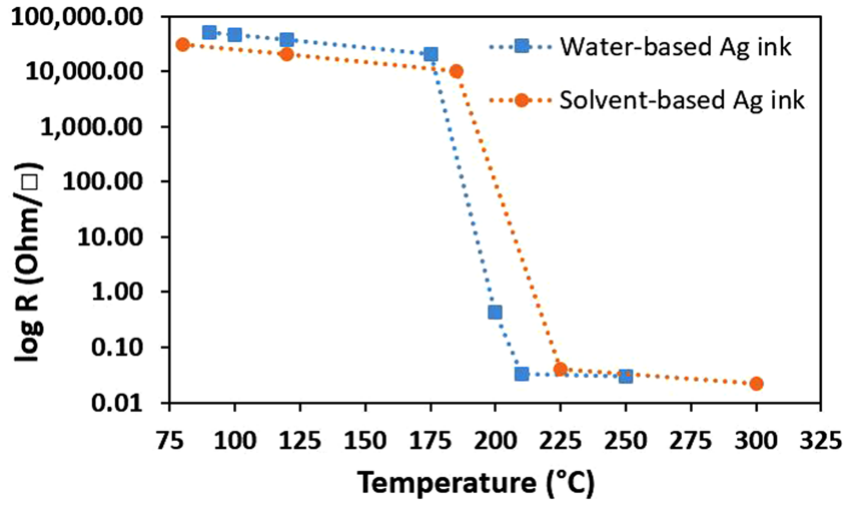
Sheet resistivity measurements of inkjet printed solvent- & water-based Ag ink traces submitted to 1-hour oven annealing. Experimental error bars are below 10% of the sheet resistivity values.

As a reference point, standard silver (Ag) traces oven-annealed for 60 minutes at temperatures up to 300 °C are first produced as control samples. The sheet resistivity as a function of the annealing temperature has been measured for water-based and solvent-based commercial silver inks (see Fig. 1). The sheet resistivity values are between 0.02–0.03Ω/□ for temperatures over 250 °C. These values are consistent with sheet resistivities provided by the commercial ink data sheets. It should be noted that only the Kapton polyimide substrate survives the high-temperature oven-annealing process without damages. Although the PET polyester substrate is rated to give excellent dimensional stability at temperatures up to 150 °C^25^, it starts showing visible signs of thermal degradation and shrinkage at oven temperatures around 200 °C. At 250 °C, the PET polyester substrate loses all mechanical flexibility and starts to crack.

### Multi-Step Dynamic Laser Sintering

**Figure 2:**
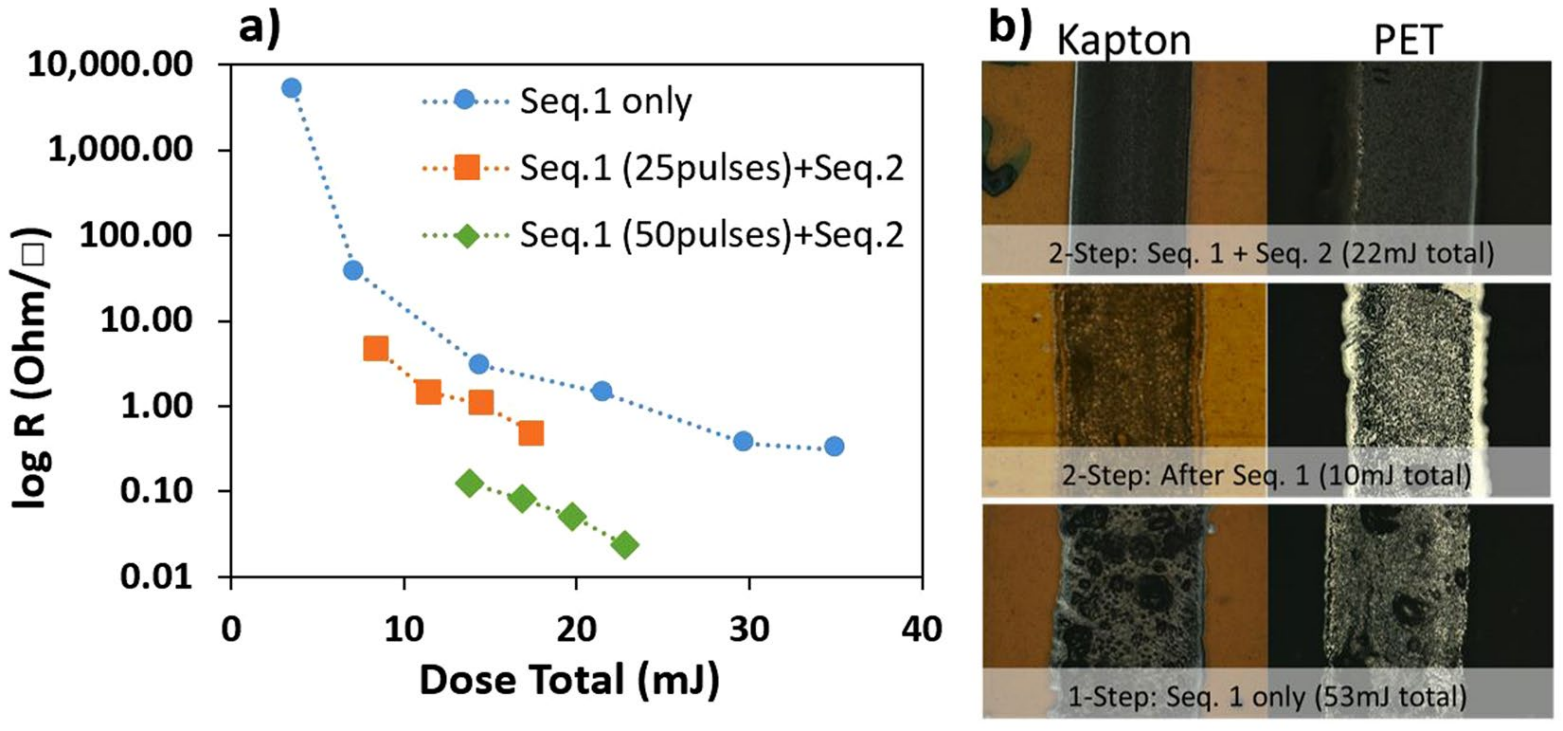
(**a**) Sheet resistivity measurements of Ag ink traces after dynamic laser sintering. Measurements error is around 20% sample-to-sample. (**b**) Sintered printed Ag traces on Kapton (left column) and PET (right column) using the 2-step method after Seq. 1 (middle row) and after Seq. 1 and Seq. 2 (top row), and comparison with results obtained using the 1-step method at a high laser dose of 0.3 mJ/pulse (bottom row).

In the first experiment, a series of 1-μs laser pulses at 1064 nm wavelength is generated by means of an INO fiber laser system to perform sintering of water-based Ag ink in different manners: a 1-step method comprising only of one laser pass, labelled «Seq. 1», and a 2-step method comprising of two independent laser passes, labelled «Seq. 1 + Seq. 2», as schematically represented in Figure S1 (Supplementary Information). The 1-step method consists of a series of 18–180 pulses (Seq. 1) of equal energies (0.2 mJ/pulse) deposited in one laser pass travelling over the sample. The 2-step method consists of 2 laser passes over the sample: the first pass (Seq. 1) with 25 or 50 pulses at a fixed energy level (0.2 mJ/pulse) and a second pass (Seq. 2) with 10–40 pulses at a higher energy level (0.3 mJ/pulse). In all cases, the laser repetition rate has been adjusted to provide different amounts of deposited energy resulting in different final total irradiation doses, while the laser traveling speed is maintained at a high velocity of 500 mm/s. The results from the sheet resistance measurements on water-based Ag ink traces after dynamic laser sintering for the 1-step method (Seq. 1) and for 2-step method (Seq. 1 + Seq. 2) are presented in Fig. 2(a).

The 1-step process is designed in an attempt to replicate the oven annealing, meaning that the ink rapidly reachs its maximum temperature, which is then maintained there for a certain curing time. Of course, the overall process remains several orders of magnitude faster. In the 2-step process design, the first laser travelling pass (Seq. 1) performed at low repetition rate to evaporate the solvent without fully sintering the Ag nanoparticles. This is consistent with the high sheet resistivities measured for the 1-step method at low repetition rates (or low dose totals) observed in Fig. 2(a). However, the second laser traveling pass (Seq. 2) performed at higher pulse energies completes the sintering of the metallic nanoparticles, resulting in significantly lower sheet resistivities. Specific laser parameters and deposited energy doses for the 1-step method and the 2-step method experiments are presented in Tables S1 and S2 (Supplementary Information), respectively. In Fig. 2(a), the results for the 2-step treatment are presented as two data sets each using a different laser repetition rate for Seq. 1. In case of the 1-step method, only a one laser pass with a 0.2 mJ/pulse energy at a repetition rate of 5–50 kHz, corresponding to doses ranging from 4–35 mJ (see Table S1 in the Supplementary Information). The final dose of 35 mJ corresponds to the highest repetition rate, which reduces the sheet resistance down to 0.3 Ω/□ (blue dots). However, this sheet resistance value remains an order of magnitude higher than the benchmark of 0.02–0.03Ω/□ obtained using the standard oven-annealing process. From these experiments, we find that a further increase of the pulse energy to 0.3 mJ/pulse in order to lower the sheet resistance causes damages due to bubbling and explosive boiling of the solvent/water content, as shown in Fig. 2(b). These damages appear at high laser repetition rates or higher pulse energies during the sintering process, which is consistent with the literature^26,27^. Here, these liquid-based phenomena appear only when using higher pulse energies of 0.3 mJ/pulse with the 1-step method. However, these damages are not observed when using pulse energies of 0.3 mJ/pulse in Seq. 2, after exposure to lower-energy pulses of 0.2 mJ/pulse (Seq. 1) in the 2-step method to evaporate the solvent. Indeed, Fig. 2(b) clearly shows more homogeneously-sintered ink traces without undesirable structural surface defects when using the 2-step method on both Kapton and PET substrates.

For the 2-step method, we also explored the effect of varying the repetition rate on the sheet resistivity of the silver ink traces. Indeed, the first laser pass (Seq. 1) with the same pulse energy (0.2 mJ/pulse) was performed at repetition rates of 7 kHz (25 pulses) or 14 kHz (50 pulses), before a a second laser pass (Seq. 2) also with the same pulse energy (0.3 mJ/pulse) and a repetition rate varying from 3–12 kHz (see Table S2 in the Supplementary Information). The two experiments using the 2-step method give sheet resistances values of 0.5 Ω/□ (Fig. 2(a) orange dots) and 0.024 Ω/□ (Fig. 2(b) green dots) for a total irradiation dose of 17 and 22 mJ, respectively. The lowest sheet resistance is not directly related to the total dose, rather to the more efficient combination of Seq. 1 and Seq. 2 together. One may note that the 1-step method (Seq. 1 total dose of 35 mJ) gives a similar sheet resistance of 0.3 Ω/□ in comparison with the 2-step method at 7 kHz repetition rate (Seq. 1 at 5 mJ dose + Seq. 2 at 12 mJ dose, 17 mJ total dose), although the deposited energy is less than half (17 mJ *vs*. 35 mJ). For the 2-step method at 14 kHz repetition rate (Seq. 1 at 10 mJ dose + Seq. 2 at 12 mJ dose, 22 mJ total dose), the sheet resistance reaches 0.024 Ω/□, which is comparable to the benchmark of 0.02–0.03 Ω/□ using standard oven annealing. Again, this low sheet resistivity is achieved with much less energy compared with using the 1-step method alone. Thus, a 2-step method appears necessary to achieve optimal sintering of silver nanoparticle-based conductive inks in aqueous solutions. This may be related to the heterogeneous nature of such inks, making thermal diffusivity and capacity properties key physical components that govern phase transitions related to the different temperature thresholds from solvent/water evaporation and solid metallic nanoparticles melting. Finally, one may explore different combinations of Seq. 1 and Seq. 2 repetition rates in order to further assess the optimization of this 2-step laser sintering process.

### Time-Domain Pulse Shaping

In the second set of experiments, 16-μs laser pulse profiles at 1064 nm wavelength are modulated in the time-domain in order to compare the effect of using square- and Gaussian-shaped laser pulses for the sintering of the solvent-based Ag inks. The INO proprietary MOPAW fiber laser allows for such unique pulse-shaping modulation, while also allowing for precise control over the pulse energies via efficient optical amplification schemes and control of nonlinear effects within the fiber core^28^. In this study, the laser repetition rate is varied in the range of 0.5–10 kHz, while preserving a constant pulse energy of 0.35 ± 0.004 mJ/pulse and a laser travelling speed of 63 mm/s. The process parameters are detailed in Table S3 (Supplementary Information).

**Figure 3:**
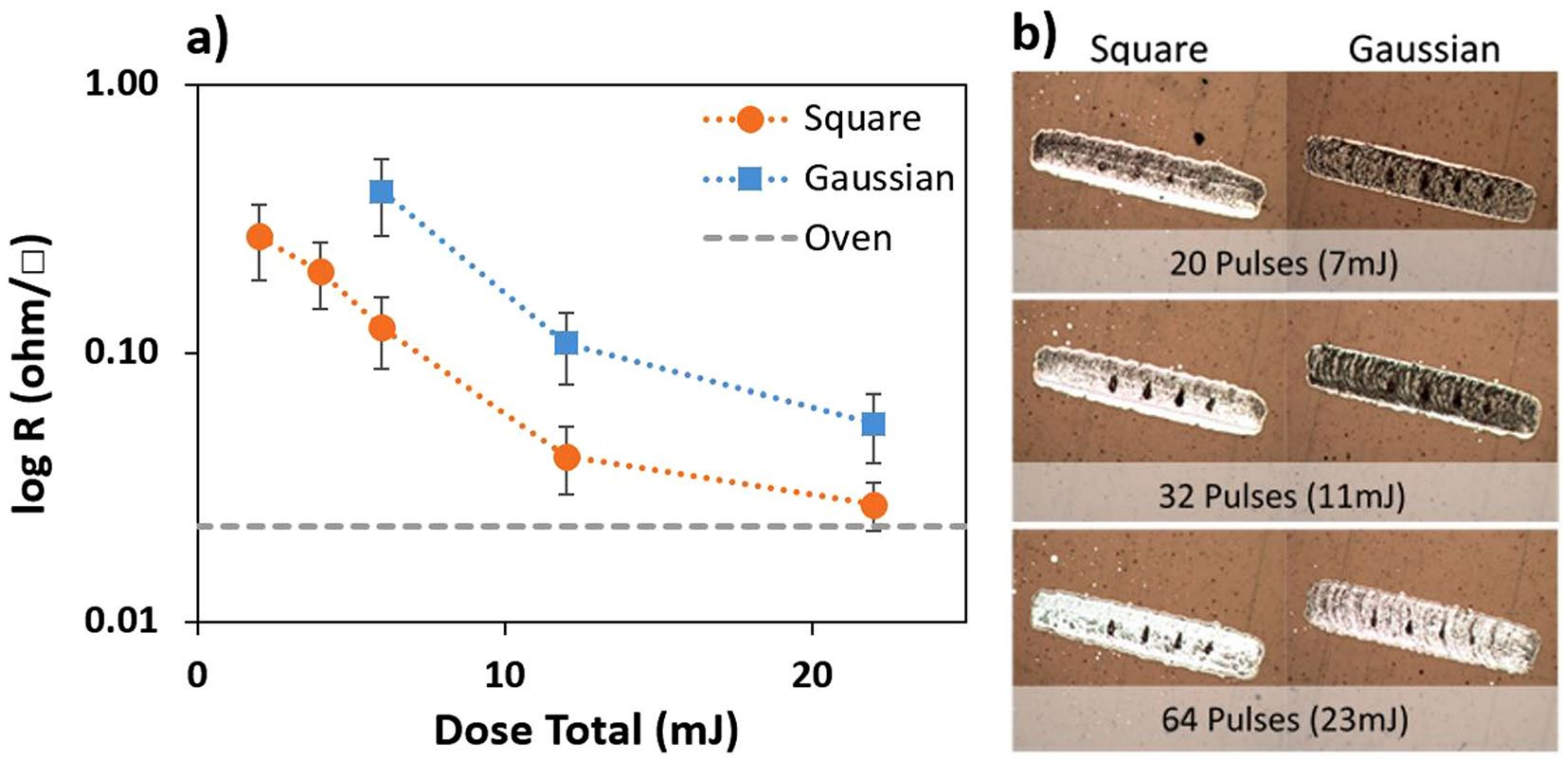
(**a**) Sheet resistivity measurements and (**b**) printed solvent-based Ag ink traces on Kapton polyimide submitted to square- and Gaussian-shaped laser sintering pulses. The reference result achieved using standard oven-annealing process is shown as dashed line. One may observe the four black dots in each traces resulting from the 4-point-probe resistivity measurements.

The sheet resistivity from laser-treated Ag ink traces on Kapton substrate as a function of the total laser energy dose are presented in Fig. 3. Square-shaped laser pulses are compared with conventional Gaussian-shaped laser pulses (in the time-domain). The benchmark 0.02Ω/□ sheet-resistivity achieved using standard oven-annealing (shown in Fig. 1) is presented as a dashed line for comparison. The optimal curing is achieved using square-shaped pulses and a laser dose of 22 mJ, resulting in sheet resistivities around 0.027Ω/□. At this maximum dose, the square-shaped pulses (Fig. 3(a) orange dots) yield a sheet resistivity as low as 0.027Ω/□, compared with 0.055Ω/□ when using Gaussian-shaped pulses (Fig. 3(b) blue dots), which can be reached using only half of the deposited energy when using square-shaped pulses.

**Figure 4:**
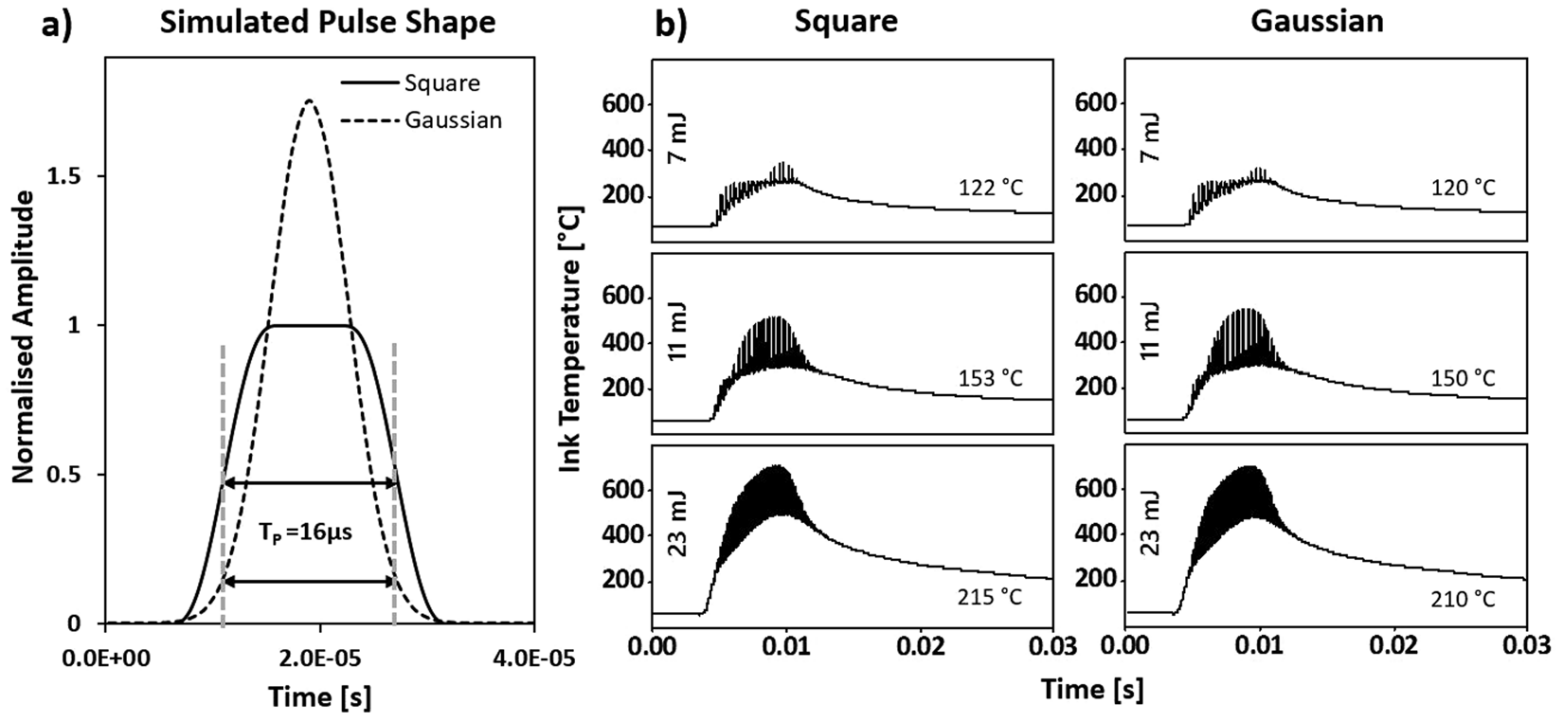
(**a**) Sketch of 16-μs square- and Gaussian-shaped laser pulses. (**b**) COMSOL modeling of the sintering process on solvent-based Ag ink traces submitted to square-shaped laser pulses (left column) and conventional Gaussian-shaped laser pulses (right column) at various laser repetition rates. The residual temperatures at 30 ms is noted for each temperature curve. Temperature curves data are taken at the center point of the ink trace.

FEM simulations of laser-mater interactions using COMSOL can provides more insight on the pulse-shaping effect on the heat-transfer processs governing the laser sintering of the conductive inks. The simulation is performed for a series of 16-μs square-shaped pulses and conventional Gaussian-shaped laser pulses, as shown in Fig. 4(a). Transient temperature curves calculated for up to 30 ms of laser exposure and different operating frequencies and taken at the center position of the the ink trace are presented in Fig. 4(b). Note that the whole process time for a 1-mm long silver trace is around 16 ms. A mix of peak and residual temperatures provides the necessary thermal energy to complete the sintering of the ink. Simulations suggest that the temperature of the Kapton substrate at 10 μm beneath the surface (corresponding to 5% of the total thickness of the substrate), follows closely the residual temperature of the sintered ink. More than 10 μm under the interface, the substrate temperature remains well below the glass transition temperature of the Kapton (360–410 °C according to the supplier’s specifications) during the whole sintering process. Top and cross-sectional views, showing internal thermal dynamics inside the ink and the substrate for the square-shaped pulse trains with a total dose of 7 mJ are presented in Video S1 (Supplementary information). These simulations represent the temperature evolution curves in a dynamic fashion for the 16 ms sintering process time plus 14 ms of thermal relaxation time. One may note the heat penetration down to only limited to the top-most part of the substrate.

**Figure 5:**
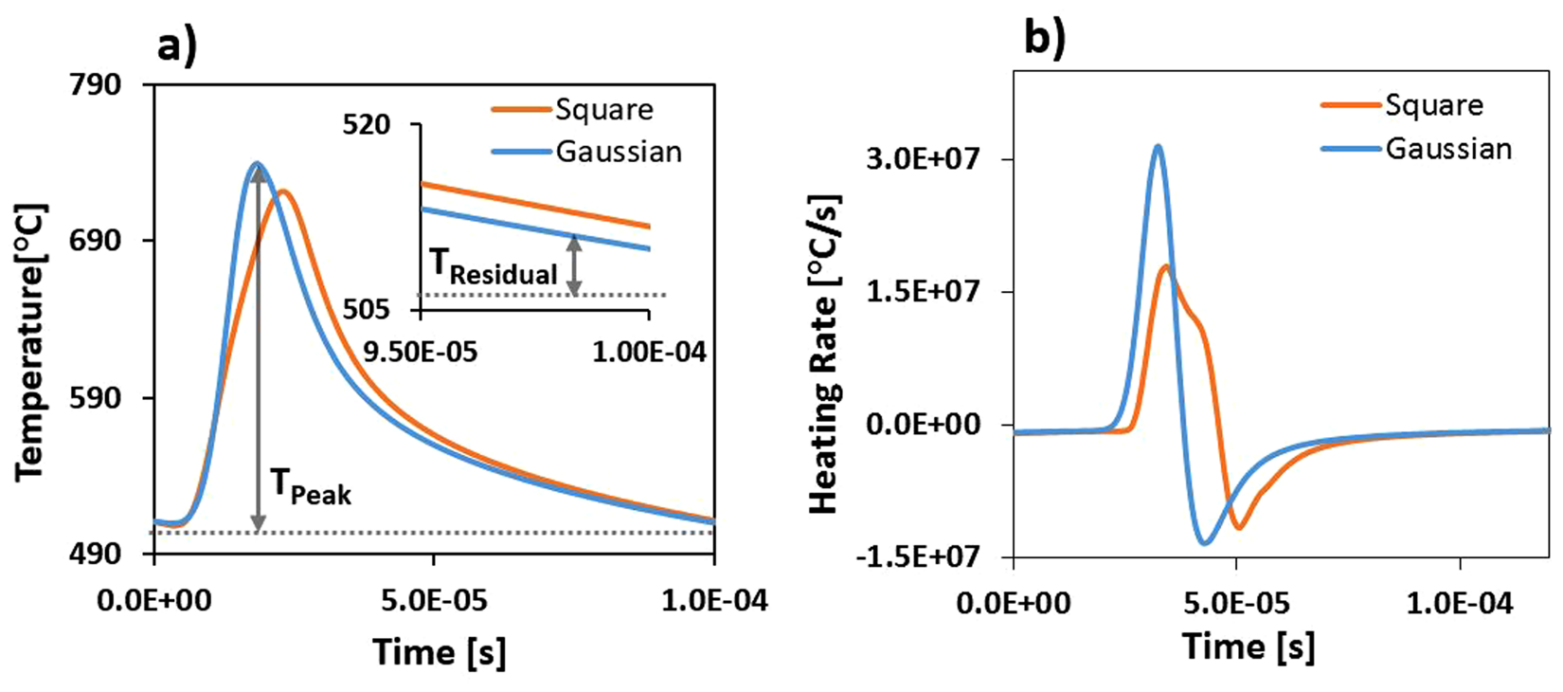
(**a**) Single-pulse temperature transients and (**b**) heating rates calculated from the derivative of the temperature transient evolutions using square- and Gaussian-shaped excitation pulses at a repetition rate of 5 kHz. The peak temperature (T_peak_) is reached at the top of the single-pulse excitation, while the residual temperature (T_residual_) is the temperature just before the next laser pulse as shown in the inset.

**Figure 6:**
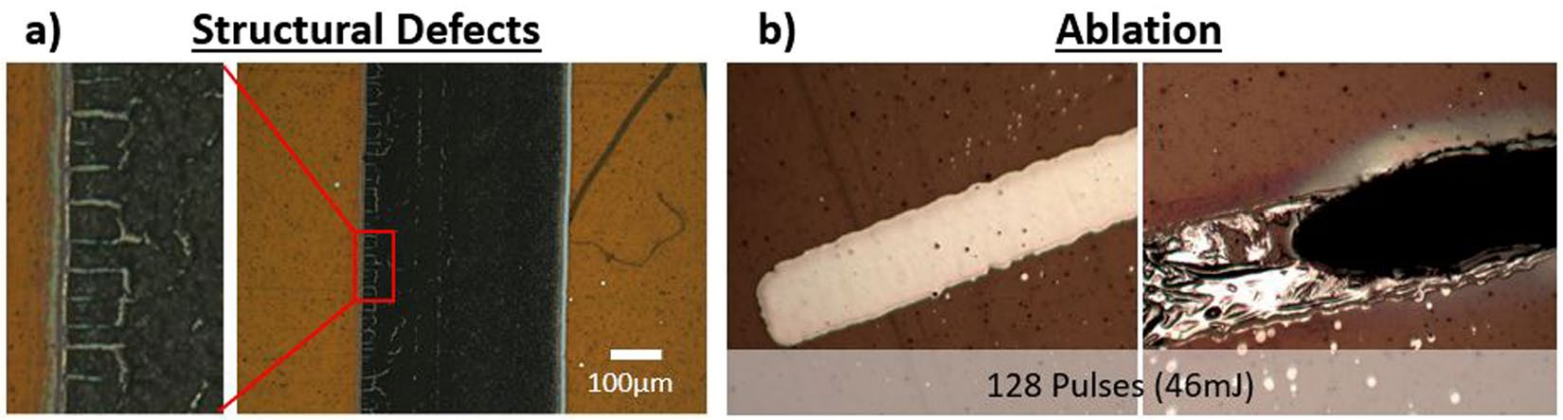
(**a**) Surface cracking due to higher thermal stress caused by higher heat exchange rates when using Gaussian-shaped excitation pulses. (**b**) Ablation phenomenon originating from overheating following laser sintering at 20 kHz (46 mJ) using Gaussian-shaped excitation pulses.

The temperature curves are highly similar for the square- and Gaussian-shaped pulses, although there is a slight difference in the maximum peak temperatures and in the final residual temperatures after 30 ms. In order to provide a deeper understandings of the experimental results, a single-pulse temperature curve is extracted from the COMSOL simulation and presented in Fig. 5(a) for reference. The peak temperature (T_peak_) is reached at the top of the impulsion and the residual temperature (T_residual_) is achieved just before the start of the next 16-μs laser impulsion. A higher temperature over a shorter time is seen at the start of the Gaussian pulse (blue line), which cools down faster and results in a lower residual temperature compared with a square-shaped pulse excitation (orange line). However, this behavior alone does not explain by itself the significant difference in the sheet resistivity values. The heating rates for single-pulse excitations using square- and Gaussian-shaped pulses are can be obtained from the time-derivative of the single-pulse temperature curves as shown in Fig. 5(b). These simulations indicate that the maximum heating rate using Gaussian-shaped excitation pulses is almost twice that of square-shaped excitation pulses at respectively 3.15 × 10^7 ^°C/s and 1.74 × 10^7 ^°C/s. These results suggest a significantly-higher laser-induced thermal stress due to both higher peak temperatures and heating rates when using Gaussian-shaped excitation pulses, which can lead to structural damage and cracking at the surface as observed in Fig. 6(a). This effect may also significantly influence the conductive properties of the sintered ink traces as well (as suggested in Fig. 3). This is consistent with the ablation evidence originating from spallation or phase explosion and observed after sintering at high repetition rates of 20 kHz using Gaussian-shaped pulses (see Fig. 6b), which doesn’t occure when using square-shaped excitation pulses. Similar phenomenon has also been observed previously in Fig. 2 when using high irradiation doses.

## Discussion

In this report, we have demonstrated that controlling time-domain energy distribution is paramount for optimizing the laser sintering process on various conductive heterogeneous nanoparticle-based silver ink formulations. By means of the INO MOPAW pulse-shaping laser system, we have shown the ability to perform rapid thermal treatment of printed inks on low-temperature susbtrates, while locally controlling the ink properties and limiting thermal penetration to the top-most printed layer.

Using a multi-step pulsed laser treatment, we observed that multiple laser passes using different pulse energies to target different phases of the sintering process significantly improves electronic properties of the conductive inks. Sheet resistivity measurements obtained using a 1-step laser sintering method are significantly higher than the benchmark values reached using standard oven annealing. Further increase of the pulse energy for this one-step laser-annealing process does not improve the sheet resistivity, but it leads to undesirable thermal damage such as surface bubbling, cracking and explosive boiling of the solvent content.

In contrast, a two-step laser annealing process results in more homogeneously-sintered ink traces with sheet resistivities comparable to the benchmark values achieved using standard oven-annealing, while preventing the undesirable structural damage. The lowest sheet resistivities achieved using the two-step method are not directly related to the highest total irradiation dose, but rather to the more efficient combination of the two laser passes. When comparing the one-step and two-step methods, one may note that the lowest sheet resistivity values reached with the one-step method be reached with only half of the total laser energy dose when using the two-step process, making it more energy efficient (35 mJ compared with 17 mJ). It appears that the first laser pass can target solvent/water-content evaporation occuring at lower temperatures, while leaving the solid content to be sintered at a higher temperature by the second laser pass, thus creating a more efficient process and a more uniformly-sintered metal trace. This process may provide better compatibility with heterogeneous ink formulations, where thermal diffusivity and thermal capacity properties are key physical components governing the phase transitions.

Moreover, we show that time-domain pulse shaping can significantly improve the conductive properties of laser-sintered metallic nanoparticle-based inks. At the maximum dose, a square-shaped pulsed laser excitation provides sheet resistivities two times lower than using conventional Gaussian-shaped excitation pulses at 0.027Ω/□ and 0.055Ω/□, respectively. In addition, the same sheet resistivities can also be reached with only half the deposited energy when using square-shaped excitation pulses. Using this process allows for a wider range of operating parameters, resulting in higher-quality sintering in terms of the energy required to achieve a specific electrical conductivity value. This approach has been found suitable for solvent-based nanoparticle conductive inks, where solvent boiling point and metal melting temperatures are closer in values. FEM modeling using COMSOL was used to further investigate and understand the effects of time-domain pulse shaping on the temperature profiles of the ink during the laser-matter interactions. These simulations support the experimental results, suggesting that controlling the heating rate during the sintering process yields more uniform ink traces without structural surface defects. Although the overall temperature profiles in the ink are highly similar when using the square- and Gaussian-shaped excitation pulses, we note a slight difference in residual and peak temperatures. When examining the heating rates seen by the ink exposed to a single laser pulse, we observe that the heating rates induced using Gaussian-shaped pulses are nearly twice those originating from a square-shaped laser pulse excitation at 3.15 × 10^7 ^°C/s and 1.74 × 10^7 ^°C/s, respectively. This heating effect may induce some undesirable thermal stress, phase explosion or spallation phenomena, which are derived from the rapid heating and cooling rates from using very high laser doses or when using Gaussian-shaped excitation pulses^29–31^. This conclusion is consistent with the higher peak temperatures, lower residual temperatures and higher peak heating rates observed from the simulations. This is also consistent with the inclusion-initiated thermal explosion (TE) model, which predicts that the laser-induced damage temperature (LIDT) for Gaussian pulses is lower than for square-shaped pulses with the same pulse width^32^. The effect of pulse-shaping clearly provides a specific interaction pathway considering the diverse nature of the materials present in ink solutions, when acting within the thermal equilibrium time of these materials. Further simulations, including mechanical stress analysis, need to be performed to further assess the validity of this phenomenon. Performing thermal dynamics FEM simulations is also a valuable tool to better identify and optimize the laser parameters before performing laser-based sintering treatment.

In conclusion, we have shown the distinct advantages of using a laser-based sintering process to provide a better control of the time-domain energy distribution using both a multi-step laser method and/or time-domain pulse shaping of the laser excitation. Both these techniques provide a greater control over the electronic properties of the resulting conducting ink traces, while providing a more energy-efficient process within rapid processing times (in the milisecond range). The FEM simulation results suggest that carefully controlling the heating rates within the ink volume is critical to avoid structural surface defects and achieve optimal resistivity values for the sintered nanoparticle-based metallic inks.

## Methodology

The conductive silver (Ag) traces submitted to laser sintering are printed using a Ceradrop F-Serie inkjet Printer with 1pL Dimatix cartridges. Two types of Ag nanoparticle inks are used: the water-based Metalon JS-B40G (40%wt. Ag) ink and the TGME-solvent-based ANP DGP 40TE-20C (30%wt. Ag) ink, respectively identified «water-based» and «solvent-based» ink throughout the manuscript. The silver nanoparticle sizes are found to be approximately 50 nm in both cases and their viscosity ranges from 10–15mPa-s for both inks. Moreover, two types of flexible substrates are used for printing: the DuPont™ Kapton® FPC polyimide and the Melinex ST505 heat-stabilized PET polyester films, which are respectively identified as «Kapton» and «PET» substrates. The sintering process is performed using the Institut National d'Optique (INO) proprietary Master Oscillator Pulsed Arbitrary Waveform (MOPAW) fiber laser system. This commercial laser platform is capable of providing customized pulse-shaping on the order of nanoseconds to microseconds with capabilities for picosecond bursts for non-thermal ablation purposes. Repetition rates up to 1 MHz are achievable with a maximum power output of 20 W at a 1064 nm operating wavelength. This wavelength is used due to its high level of aborption by the conductive Ag traces (Fig. S3(a) in the Supplementary Information). An optical attenuator is used to control the power output of the laser. The average power of the laser has been measured through a calibrated Gentec thermopile power detector. The samples are positioned on Intelligent Actuator stage with an X-Y displacement rails, having a traveling speed up to 600 mm/s calibrated by means of tachymetry, in order to allow for dynamic sintering process. The stage is heated with a thermoelectric cooler (TEC) at a constant 60 °C to reflect the chuck temperature used during printing. Sheet resistivity measurements are taken using a conventional 4-point-probe apparatus and averaged over 10 samples for each set of test parameters. Mechanical integrity testing of the sintered Ag traces is achieved by means of 3 M MIL-A-AA-113-B (formerly L-T-90) Type 1 Class A adhesion tape-test (on-going study). Finally, standard 1-hour oven annealing of printed Ag traces at temperatures up to 300 °C is performed in order to provide a comparative benchmark for the laser sintering. Finite elements method (FEM) simulations of microsecond-pulsed laser-matter interactions on solvent-based Ag ink has been developed using COMSOL Multiphysics® Version 5.2 and makes use of the Heat-Transfer and Materials Library Modules.

### Time-Domain Pulse Shaping & Multi-Step Dynamic Pulsed Laser Sintering

In this study, two different sets of experiments are performed. In the first set, 1 μs-pulses in the range of 3–84 kHz are used, using a 1.8 × 1.8 mm^2^ laser beam spot with a traveling speed of 500 mm/s over 1 mm-wide and 1 μm-thick printed water-based Ag traces. In the second set, 16 μs-pulses in the range of 0.5–10 kHz are used, using a 0.4 × 0.4 mm^2^ laser beam spot with a traveling speed of 63 mm/s over 400 μm wide and 0.5 um thick printed solvent-based Ag traces. For this set of experiments, a customized square-shaped waveform and a conventional Gaussian-shaped pulse are used and identified as «Square» and «Gaussian» in Fig. 4(a), and their effects on the laser sintering process are investigated. An optical flat-top laser beam shaper is used in order to provide a spatially-uniform deposited energy distribution on the samples. The measured laser beam shape has been performed using a NIR imaging camera from Spiricon (Fig. S2 Supplementary Information). In both series, a total dose ranging from 22–35 mJ is necessary to obtain an efficient sintering process, giving valuable low sheet resistivity values. The total dose is calculated by taking the energy per pulse (E [J/pulse]), which is obtained from the ratio of the average measured power (P [W]) to the repetition rate of the laser (RR [Hz]), multiplied by the number of pulses (N [pulse]). This last number is calculated from the ratio of the laser beam spot diameter (d [mm]) to the scanning speed (v [mm/s]), multiplied by the repetition rate of the laser.

### Time-Domain Pulse Shaping and FEM Simulations

Time-dependent FEM simulations of the pulsed-laser sintering process on heterogeneous solvent-based silver ink used in the second series of experiments are performed using COMSOL modeling to acquire a better understanding of the effects of time-domain pulse-shaping. A printed 500 nm-thick trace with lateral dimensions of 1 mm (length) by 400 μm (width) is modelled as a bulk heterogeneous material, where its temperature-dependent material properties are either estimated by a weighted average of the materials composition properties, acquired experimentally or by taking typical values from literature examining similar ink material compositions^33,34^. Two different phase transitions are integrated within the model as an heterogeneous material; a liquid-gaseous phase transition for the solvent content of the ink and a solid-liquid phase transition for the silver content that are estimated to happen at 255 °C and 200 °C, respectively. The ink trace is supported by a flexible 200 μm thick Kapton polyimide substrate with lateral dimensions of 3 mm × 2 mm, large enough to simulate an infinite substrate while minimizing simulation time. The bottom surface of the substrate is modeled to have a constant temperature of 60 °C to mimic the printer chuck acting as a heat sink. The material properties of the solvent-based Ag ink and the supporting Kapton polyimide substrate and taken from the supplier’s data sheet^35^ are presented in Table S4 (Supplementary Information).

The pulsed laser is modelled as a heat flux on the surface of the silver ink trace, as the non-reflected portion of the laser beam energy is assumed to be fully absorbed within the first few nanometers below the surface. The pulsed-laser heat flux equation is shown in Equation 1 and contains four key components: a constant tied to the laser power (P), a temperature-dependent absorption coefficient (A(T)), a spatial- and time-dependent beam parameter to account for the shape and displacement of the laser beam (Beam(x, y, t)), and a time-dependent pulse train parameter to account for the pulsed nature of the laser (PulseTrain(t)).

1 $$PulsedLaser(x,y,t,T)=P\cdot A(T)\cdot Beam(x,y,t)\cdot PulseTrain(t)$$

The absorption parameter is modelled as a piecewise function and has been assessed from experimental results obtained by means of spectrophotometry from optical reflectivity and transmission measurements of the solvent-based ink traces as a function of various oven sintering temperatures ranging from 75–300 °C. The reflectivity and transmission measurements as well as the absorption parameter as a function of temperature are shown in Fig. S3(b) in the Supplementary Information. The laser beam parameter is modelled as a moving square flat-top beam centered at x = 0 using Equation 2, where w_x_ and w_y_ are the half-width dimensions of the beam in the x and y directions, respectively (meaning that for a beam of 400 μm × 400 μm, w_x_ = w_y_ = 200 μm), y_0_ is the starting position of the beam, and V_y_ is the beam velocity in the y direction.

2 $$Beam(x,y,t)=\,{e}^{-(2{(\frac{x}{{w}_{x}})}^{10}+2{(\frac{y-({y}_{0}+{V}_{y}\cdot t)}{{w}_{y}})}^{10})}$$

The time-dependent pulse train parameter simulates the square-shaped and Gaussian-shaped laser pulses, as well as the pulsed nature of a flat-top laser beam. For the Square pulse, the pulse train is modelled using the square waveform function in COMSOL. The Gaussian pulse train is produced by introducing a periodicity to the COMSOL Gaussian pulse function. The pulse width of 16 μs is taken as the FWHM of the Square pulses and the as the FWTM (Full Width Tenth-Height) of the Gaussian pulses, mimicking the laser pulses produced by the MOPAW system used in the experiments. The laser power constant (P) varies depending on the pulse shape. For the COMSOL Gaussian pulse function, the integral of each pulse is equal to unity, meaning that the constant P is simply the value of the total pulse energy (350 μJ). Whereas it is the amplitude of each pulse for the square pulse train which is equal to unity, therefore the constant P is the pulse energy divided by the pulse width (350 μJ/t_p_). The mesh size and time steps are important parameters in any time-dependent FEM simulations, they need to be small enough to simulate the physico-chemical interactions, while keeping them large enough to minimize computation time. In our case, a refined mesh is used to accurately model the heat transfer from the laser to the ink within the studied volume. In contrast, a larger mesh size is used for the substrate since it is shown to sustain little temperature variation and thus does not need to be spatially-resolved as accurately^36^. A time-step upper limit of 1.6 μs (t_p_/10) is used to achieve a higher precision regarding the laser pulses, while keeping the computation time to a minimum. Larger time-step limits are avoided since they may affect the accuracy of the modelled Gaussian pulses, truncating the top-most sections of some of the pulses in the pulse train.

## Electronic supplementary material

Supplementary Information

Video S1

Video S2
