# Supplementary material for: Thermal Dynamics Effects using Pulse-Shaping Laser Sintering of Printed Silver Inks
Source: Sci Rep. 2018 Jan 23;8:1418. doi: 10.1038/s41598-018-19801-4 (PMC5780432; doi:10.1038/s41598-018-19801-4)
Supplement: Supplementary file 1 — Supplementary Information [file 41598_2018_19801_MOESM1_ESM.docx]

**Thermal Dynamics Effects using Pulse-Shaping Laser Sintering of Printed Silver Inks**

M. Bolduc^a*^, C. Trudeau^a,b^, P. Beaupré^a^, S.G. Cloutier^b^ and P. Galarneau^a^

^a^Institut National d’Optique, 2740 Einstein Street,

Québec, QC, Canada G1P 4S4

^b^Department of Electrical Engineering, École de Technologie Supérieure,

1100 Notre-Dame Ouest,

Montréal, QC, Canada H3C 1K3

*Correspondence to [martin.bolduc@ino.ca](mailto:martin.bolduc@ino.ca)

**SUPPLEMENTARY INOFRMATION**


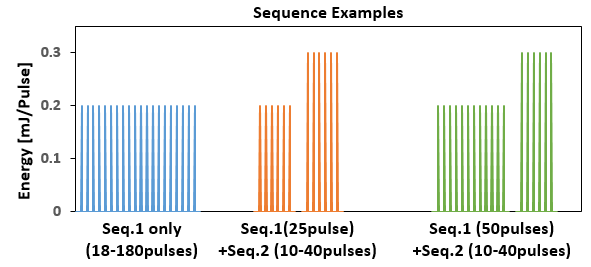


Figure S1. Schematic representation of pulse sequence combinations for 1-step (Seq.1) & 2-step (Seq.1+Seq.2) methods used in dynamic pulsed laser sintering.

*
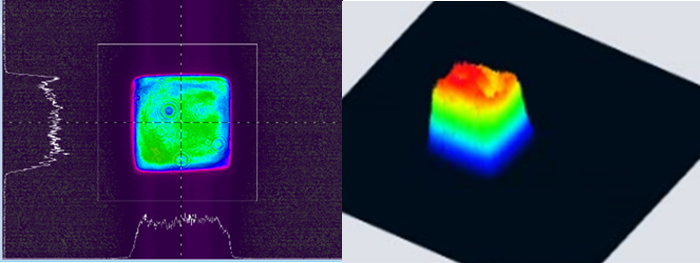
*

*Figure S2. Spatially uniform squared flat-top laser beam*


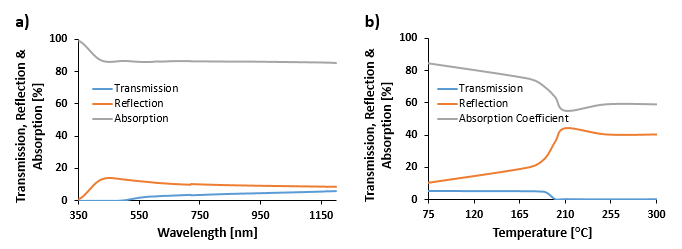


*Figure S3. (a) Transmission, Reflection and Absorption spectra of unsintered solvent-based ANP DGP 40TE-20C Ag conductive ink trace. (b) Optical Reflectivity and Transmission measurements at 1064nm of solvent-based ANP DGP 40TE-20C Ag conductive ink at oven sintering temperatures in the range of 75-300°C and associated temperature-dependent absorption coefficient.*

*Table S1: 1-step method pulsed-laser sintering sequence parameters (Seq.1)*

| **1-Step; 1μs pulse width; 1.8X1.8mm^2^ beam size;** | | | | **Laser Speed:**  **500 mm/s** |
| --- | --- | --- | --- | --- |
| **1-mm wide and 1-μm thick printed water based Ag traces** | | | |  |
| **Seq. 1: 0.2mJ/pulse** | | | | |
| Repetition Rate  [kHz] | Power  [W] | Duty Cycle  [%] | Number of Pulses | Dose  [mJ] |
| 5 | 1 | 0.5 | 18 | 4 |
| 10 | 2 | 1.0 | 36 | 7 |
| 20 | 4 | 2.0 | 72 | 14 |
| 30 | 6 | 3.0 | 108 | 22 |
| 40 | 8 | 4.0 | 144 | 30 |
| 50 | 10 | 5.0 | 180 | 35 |

*Table S2. 2-step method pulsed-laser sintering sequence parameters (Seq.1+Seq.2)*

| **2-Step; 1μs pulse width; 1.8X1.8mm^2^ beam size;** | | | | | | | **Laser Speed:  500 mm/s** | | | |
| --- | --- | --- | --- | --- | --- | --- | --- | --- | --- | --- |
| **1-mm wide and 1-μm thick printed water based Ag traces** | | | | | | |  |  |  |  |
| **Seq. 1: 0.2mJ/pulse** | | | | | **Seq. 2: 0.3mJ/pulse** | | | | |  |
| Repetition Rate  [kHz] | Power  [W] | Duty Cycle  [%] | Number of Pulses | Dose  [mJ] | Repetition Rate  [kHz] | Power  [W] | Duty Cycle  [%] | Number of Pulses | Dose  [mJ] | **Total Dose [mJ]** |
| 7 | 1.5 | 0.7 | 25 | 5 | 3 | 1 | 0.3 | 10 | 3 | **8** |
| 7 | 1.5 | 0.7 | 25 | 5 | 6 | 2 | 0.6 | 20 | 6 | **11** |
| 7 | 1.5 | 0.7 | 25 | 5 | 9 | 3 | 0.9 | 30 | 9 | **14** |
| 7 | 1.5 | 0.7 | 25 | 5 | 12 | 4 | 1.2 | 40 | 12 | **17** |
| 14 | 3 | 1.4 | 50 | 10 | 3 | 1 | 0.3 | 10 | 3 | **13** |
| 14 | 3 | 1.4 | 50 | 10 | 6 | 2 | 0.6 | 20 | 6 | **16** |
| 14 | 3 | 1.4 | 50 | 10 | 9 | 3 | 0.9 | 30 | 9 | **19** |
| 14 | 3 | 1.4 | 50 | 10 | 12 | 4 | 1.2 | 40 | 12 | **22** |

*Table S3 Pulse-shaping laser sintering sequence parameters*

| **16μs pulse width; 0.4X0.4mm^2^ beam size;** | | | | **Laser Speed:**  **63 mm/s** |
| --- | --- | --- | --- | --- |
| **400-μm wide and 0.5-μm thick printed solvent based Ag traces** | | | |  |
| **0.35mJ/pulse** | | | | |
| Repetition Rate  [kHz] | Power  [W] | Duty Cycle  [%] | Number of Pulses | **Dose  [mJ]** |
| 0.5 | 0.2 | 0.8 | 3 | **2** |
| 1.5 | 0.5 | 2.4 | 10 | **4** |
| 3.0 | 1.0 | 4.8 | 20 | **8** |
| 5.0 | 2.0 | 8.0 | 32 | **12** |
| 10.0 | 4.0 | 16.0 | 64 | **22** |

Table S4. Temperature-dependent material properties of the simulated solvent-based Ag ink and polyimide substrate

| **Material Properties used in FEM simulation** | | | |
| --- | --- | --- | --- |
| **Property** | **Solvent-based Ag Ink** | **Kapton Substrate** | **Units** |
| Thermal Conductivity | 0.036e^(0.0153[1/K]*T)^ (0-327°C) [32] 420 (≥327°C) [33] | 0.12 [34] | W m^-1^ K^-1^ |
| Heat Capacity | 2.09 (0-255°C)*  0.24 (≥255°C) | 1.09 [34] | J g^-1^ K^-1^ |
| Density | 1560 | 1420 [34] | Kg m^-3^ |
| Latent heat of melting | 26.4 (200°C)* | - | kJ kg^-1^ |
| Latent heat of evaporation | 560 (255°C)* | - | kJ kg^-1^ |
| Initial temperature | 60 | 60 | °C |

*Weighted average from bulk Ag and bulk ethylene glycol values

*Video S1 & S2. (See video files) Thermal dynamics results from the shaped Square pulse train sintering simulation, with a total dose of 7mJ. (S1) Top view. (S2) Cross-sectional view. Simulation data shown for 30ms (16ms sintering time + 14ms cooling time). Image Snapshots taken from the Videos at 0.016s.*
