# Supplementary figures and images for: Thermal Dynamics Effects using Pulse-Shaping Laser Sintering of Printed Silver Inks
Source: Sci Rep. 2018 Jan 23;8:1418. doi: 10.1038/s41598-018-19801-4 (PMC5780432; doi:10.1038/s41598-018-19801-4)

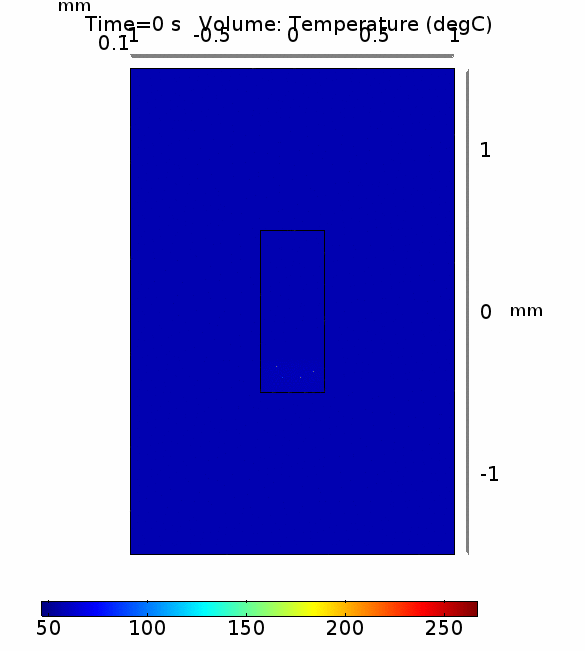

Supplement: Supplementary file 2 — Video S1 [file 41598_2018_19801_MOESM2_ESM.gif]

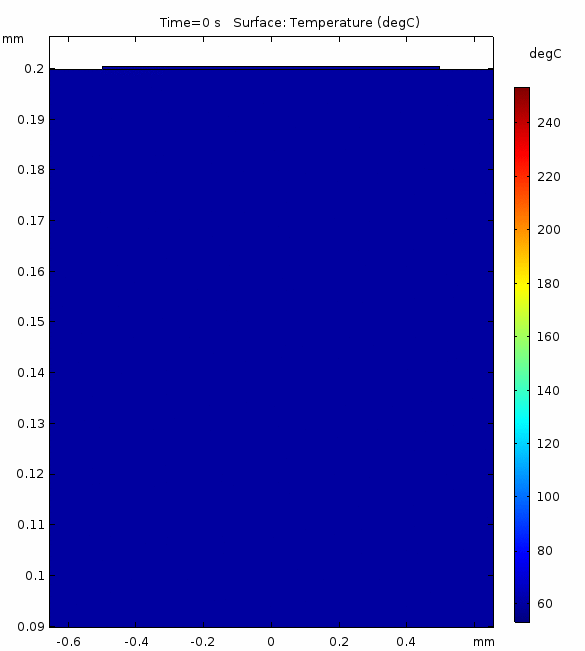

Supplement: Supplementary file 3 — Video S2 [file 41598_2018_19801_MOESM3_ESM.gif]
